# An Old Defence Against New Infections: The Open-Air Factor and COVID-19

**DOI:** 10.7759/cureus.26133

**Published:** 2022-06-20

**Authors:** Richard Hobday, Peter Collignon

**Affiliations:** 1 Public Health, Hobday Research, Cwmbran, GBR; 2 Microbiology, Canberra Hospital, ACT Health, Canberra, AUS; 3 Medical School, Australian National University, Canberra, AUS

**Keywords:** infection control, biodefence, influenza, covid-19, ventilation, open air factor, sanatorium, open-air regimen, florence nightingale

## Abstract

Outdoors, the risks of transmission of COVID-19 and many other respiratory infections are low. Several environmental factors are known to reduce the viability of viruses and other infectious pathogens in the air. They include variations in temperature, relative humidity, solar ultraviolet radiation, and dilution effects. But one agent that reduces the viability of both viruses and bacteria outdoors, the germicidal open-air factor (OAF), has not been properly recognized for decades. This is despite robust evidence that the OAF can influence both the survival of airborne pathogens and the course of infections.

The germicidal effects of outdoor air were widely exploited during the late 19th and early 20th centuries. Firstly, in the treatment of tuberculosis patients who underwent 'open-air therapy' in sanatoria; and secondly by military surgeons during the First World War. They used the same open-air regimen in specially designed hospital wards to disinfect and heal severe wounds among injured soldiers. It was also used on influenza patients during the 1918-19 pandemic. Later, in the 1950s, open-air disinfection and treatment of burns were proposed in the event of nuclear warfare. During the 1960s, the OAF briefly returned to prominence when biodefence scientists conducted experiments proving that open air has a potent germicidal effect. When this work ended in the 1970s, interest in the OAF again fell away, and it remains largely ignored.

The COVID-19 pandemic has revived interest in understanding the transmission dynamics and survival of viruses in the air. The pandemic has also stimulated research in the science and practice of improved ventilation to control respiratory infections. Such work is incomplete without an appreciation of the inactivation of viruses and other pathogens by the OAF, but this needs further investigation as a matter of urgency. Research to better understand the conditions under which the OAF can be preserved indoors is urgently needed. We need to review building design with better regard to infection control and patient recovery. But we need to act without delay, as there is already sufficient evidence to show that public health generally would improve if more emphasis was placed on increased exposure to outdoor air.

## Introduction and background

Background

As the COVID-19 pandemic has unfolded, it has become clear that person-to-person transmission occurs relatively rarely when people are outdoors [1,2]. By contrast, infection risk appears to be greatly increased in poorly ventilated indoor spaces [3-6]. In common with many other respiratory diseases, contracting COVID-19 is most often an 'indoor event'. The greater and quicker dilution and dispersal of infectious particles outdoors play a major part, but other factors contribute. Variations in temperature and relative humidity can inactivate coronavirus in the environment [7]. Solar ultraviolet radiation inactivates coronaviruses and does so rapidly [8-10]. The potential contribution of the open-air factor in the inactivation of coronavirus could be equally important. But this has not been investigated, despite robust evidence of its viricidal action.

For close to two hundred years, there have been reports that 'fresh air' protects against respiratory infections because it is germicidal. There is robust scientific evidence in support of this. So, could this be a major factor in protecting people from contracting COVID-19, especially when they are outdoors?

The open-air factor

In 1968, the journal Nature published a communication entitled 'Unstable germicidal pollutant in rural air'. This was a report of experiments in which outdoor air was shown to be more lethal to airborne pathogens than indoor air [11]. Scientists at the Microbiological Research Establishment at Porton Down, Wiltshire, had developed a new technique to measure the effects of outdoor air on the survival of bacteria, viruses, and spores: they suspended them on ultra-fine threads stretched across small metal frames. This created a form of 'captive aerosol' [12]. The metal frames were held in a position, which protected the micro-threads from rain and gusting winds but allowed the free exchange of air. In the first experiments, the survival of the bacterium Escherichia coli (E. coli) outdoors was compared to that of samples in a closed vessel containing clean air at the same humidity and temperature as the open air [11,12]. Tests were initially carried out during the hours of darkness as, in common with other bacteria and viruses, E. coli are rapidly killed by sunlight [13]. The E. coli samples exposed to outside air usually died off rapidly, but not so indoors. On some occasions, the E. coli samples in free air lost viability in 30 minutes, whereas those in enclosed air survived for several hours [13]. The bactericidal effect varied from night to night, and it disappeared rapidly in any form of enclosure. It also disappeared when the air was passed down a tube. The optimum air velocity over micro-threads at the mouth of a tube was about four miles per hour. At slower air speeds, the germicidal action was reduced [11].

Results indicated the chemical agent responsible for the bactericidal effect was stable in free air but decomposed on contact with surfaces and that the chemical nature of the surface influenced the rate of decomposition [11]. However, identifying the germicidal component of outdoor air, which the researchers referred to as the open-air factor, proved problematic. Laboratory tests in which E. coli samples were exposed to ozone levels normally present in rural air had no effect. Neither did nitrogen oxides (NOX ), sulfur dioxide (SO_2_), formaldehyde (HCHO), and ionized air. In addition, fluctuations in temperature and humidity were ruled out. The scientists involved concluded: 'The properties of the OAF do not conform to any of the commonly reported pollutant gases or to ozone' [11].

The OAF's germicidal effect was not specific to E. coli as samples of Brucella suis, Francisella tularensis, Staphylococcus epidermidis, a group C Streptococcus and Serratia marcescens lost viability at similar rates [14]. Studies with viruses showed these were extremely sensitive and decayed at higher rates than E. coli. The toxicity of OAF to bacteria varied. By contrast, the viricidal effect stayed constant throughout [15]. Bacterial spores were not sensitive. They retained their viability through the hours of darkness [14].

At the time of these tests, no chemical technique was sensitive enough to identify and measure the OAF present in the air. The ephemeral nature of the OAF in the experimental apparatus made it hard to isolate. By the 1970s, scientists had concluded that the OAF was not a single molecule [15] but a mixture of highly reactive chemical species which varied in composition [16]. The authors of a 2021 review paper on the subject supported this position, arguing that no single molecule or class of molecules can achieve the level of bactericidal activity reported in the Porton Down experiments [17]. One potential component of the OAF, the hydroxyl radical (HO), has been generated artificially and used to kill airborne pathogens [18-21]. However, the experts in atmospheric science who reviewed the available evidence in 2021 concluded that HO radicals are not directly responsible for the potent germicidal effects of the OAF. Instead, a range of products - each acting as distinct germicides - appear to be combining and contributing to the OAF. But they conceded that 'the compounds responsible for the OAF remain a mystery' [17]. This may explain why the OAF continues to be neglected in public health and infection control.

The forgotten history of the OAF

Perhaps understandably, the Ministry of Defence scientists who devised the term OAF appear to have been unaware of earlier research on fresh air's germicidal properties and of the open-air regimen [22]. During the last decades of the 19th century, fresh air was thought capable of killing pathogens such as streptococci, staphylococci, and Mycobacterium tuberculosis [23]. The air from pine forests was believed to be particularly effective [24]. The idea that the atmosphere of evergreen forests holds health benefits for people suffering from tuberculosis (TB) is old. Pliny the Elder (AD 23-79) recommended breathing it, arguing pine trees imparted a property to the air of forests, which was an effective remedy for pulmonary TB and facilitated recovery after a long illness [25]. In the 1880s, Dr. Alfred Loomis, a pioneer of TB therapy in the United States, held that it was an 'acknowledged fact' among the medical profession that pine forests had a beneficial effect on TB. In his view, clinical experience had established this 'beyond question' [26]. It is for this reason that the preferred location for TB sanatoria was often among or near pine trees [27].

In his writing on the subject, Loomis appears to have identified what, some 80 years later, came to be known as the OAF. Ozone is present in large quantities in evergreen forests [28]. This is a germicidal agent. But Loomis stated there was another antiseptic element in them. The vapor given off by pine trees combined with ozone produces an airborne disinfectant. This was not only lethal to germs but also beneficial to health. The antiseptic element in forest air was both a 'stimulant and tonic' to normal physiological processes within the lung. He thought the most likely candidate for this disinfectant was hydrogen peroxide or some other organic oxide. Dr. Loomis concluded it should be possible to make the air of houses antiseptic by growing evergreen trees nearby [26]. Doctors and hospital architects also placed great value on building sanatoria in areas where there was an abundance of ozone, believing it was this oxidant that purified the air the tuberculous patients breathed and, in doing so, helped them recover [23]. In an experiment from 1894, a current of air was reported to have a disinfecting effect on tuberculous sputum, even in the dark. By contrast, when no fresh air was present or in confined air, Mycobacterium tuberculosis retained its infectivity for long periods [29]. Little further research appears to have been published to support this work, until that of the British Ministry of Defence in the 1960s [11].

## Review

The open-air regimen

By the first decade of the 20th century, there was a widespread belief among the medical profession that fresh air could cure pulmonary tuberculosis. The pioneer in this field was Dr. George Bodington who published an essay in 1840 in which he set out how he had used fresh rural air, gentle exercise in the open, and a nutritious diet to heal patients with the condition. This was at a time when the disease was considered incurable in medical circles [30]. Subsequently, Florence Nightingale became an advocate of pure air in sickrooms based on her experiences of nursing during the Crimean War (1854-6) [31]. She stipulated that the first canon of nursing was that air indoors had to be as fresh as it was outside. Florence Nightingale state: 'Always air from the air without, and that, too, through those windows, through which the air comes freshest' [32]. To this end, Nightingale promoted the 'pavilion plan' hospital: an arrangement of separate ward units designed for cross-ventilation - during the daytime and at night - through open windows [33]. The pavilion ward and, later, the tuberculosis sanatorium were two of the first building types specifically designed to take advantage of the purported recuperative and germicidal properties of fresh air. They were also designed to admit sunlight, as solar radiation was known to be a natural disinfectant - even through glass [34]. The regimen also informed school design. Open-air schools were part of the public health campaign against tuberculosis and other childhood diseases. Like tuberculosis wards, such schools had windows that folded back along one or more walls. These opened onto terraces or verandahs for lessons outside [35].

The First World War, influenza, and nuclear war

The 'open-air' regimen became the mainstay of tuberculosis therapy and, during the First World War (1914-1918), was discovered to be an effective treatment for wounds. This occurred in the early weeks of the conflict when injured soldiers began to suffer virulent wound infections. Many soldiers were nursed outdoors because the smell of their septic and often gangrenous wounds was too offensive to tolerate in hospital wards. A British surgeon found that putting patients outside and then leaving their infected wounds open to fresh air greatly improved recovery: 'The results were almost magical, for in two or three days the wounds lost their odor and began to look clean, while the patients lost all signs of the poisoning which had been so marked before' [36].

The practice proved so successful that special wards were designed for the open-air treatment of infected wounds and for the general infections that often accompanied them [37,38]. Also, during the 1918-1919 influenza pandemic, patients nursed outdoors are reported to have recovered in greater numbers than those in hospital wards [39].

During the 1950s, there was renewed interest in open-air therapy for the casualties of warfare. On this occasion, the regimen was proposed for the mass treatment of burns in the event of nuclear war [40]. Under such disaster conditions, adequate numbers of dressings and the facilities for their use were unlikely to be available [41]. The open-air regimen was considered the only viable treatment. Control of infection was considered to be the 'outstanding feature' of this approach [41]. Nevertheless, in the years that followed, the regimen fell into disuse.

Ventilation: can the OAF be preserved indoors?

Significantly, the OAF research at Porton Down in the 1960s and 70s showed the germicidal properties of outdoor air could be fully retained in an experimental container if ventilation rates were kept high enough [42,43]. The scientists at Porton developed a ventilated sphere system that allowed them to measure the survival of airborne pathogens in conditions that were equivalent to the open air. The equipment consisted of a seven-meter diameter mild-steel sphere with four extract fans on the roof. The fans assisted natural ventilation by producing a maximum of 28 air changes per hour (ACH) [42].

The sphere also had a by-pass tube from which aerosol samples could be withdrawn. This was also used to expose animals to hazardous airborne micro-organisms [42]. The aim was to find out if a relationship existed between the viability of a given airborne pathogen and its virulence once inhaled. During tests with the category IV pathogen Francisella tularensis, guinea pigs were held for three weeks following exposure, and any deaths recorded. The results showed that OAF exposure reduced both the viability and also the virulence of the bacterium. One conclusion drawn from this was that the potential of Francisella tularensis as a biological weapon was significantly reduced by the OAF [44]. Tests were also carried out on the influenza virus, to which mice were exposed to aerosols. The 'outdoor air' in the container was markedly viricidal. This finding supported the idea that the risk of catching influenza indoors is far higher than outside [45].

The minimum rate of fresh air for full preservation of OAF in the sphere was about 12 ACH. Results from various rates of ventilation suggest that the half-life of the OAF was about three minutes in the sphere [42]. Tests were then carried out with different types of containers covering a wide range of sizes. The minimum ventilation rate needed to fully preserve the toxic properties of open air was found to be proportional to the ratio of the surface area of each vessel to its volume. The minimum ACH which fully preserved the OAF were: in the sphere, 12.5-13 ACH; in a cuboid and cube, 30-36 ACH; in a large tube, 240-360 ACH; and a small-bore tube, 3500-5000 ACH [43]. Significantly, the minimum rates needed to preserve the OAF in the cube and cuboid containers are comparable to ventilation rates measured in cross-ventilated hospital wards. Following the 2003 SARS outbreak, case studies indicated that cross-ventilation is an effective way of controlling SARS infection in hospital settings [46]. Equally, a study of ventilation and infection rates in different rooms occupied by tuberculosis patients found that pre-1950 hospitals with high ceilings and large windows offer better protection than more modern designs with lower ventilation rates. The older wards allowed ventilation rates of 40 ACH [47]. How much this was simply due to high ventilation rates diluting and dispersing tuberculosis in the air, compared to OAF killing effects as well, remains uncertain.

Discussion

During the pre-antibiotic era, hospitals and sanatoria were often built to exploit the germicidal and health effects of rural air. They were designed for high ventilation rates, both during the day and at night. Patients were encouraged to breathe cold, pure air, as this was thought to help them recover. Rural air was also held to be important in controlling the spread of infections indoors and disinfecting wounds. In the 1960s, the lethality of rural outdoor air to airborne pathogens was confirmed at Porton Down. Significantly, the scientists who carried out this work did not think the germicidal effect was due to a single molecule. Rather, they believed it to be due to a mixture of unidentified reactive chemicals. A recent review of the evidence reached the same conclusion [17]. While the causative agents have yet to be appropriately identified, the most likely candidates that contribute to the OAF may be a range of distinct germicides which are formed when alkenes in the air react with ozone. If so, Dr. Loomis appears to have been correct in proposing the ozonolysis of terpene in his paper on the subject back in 1887.

Some of the pathogens tested at Porton Down in the 1960s pose a greater threat to global public health than they did at the time because of increasing antimicrobial resistance [48]. The public health community is also concerned about the next influenza pandemic and new, virulent micro-organisms [49]. Faced with these threats to global public health, including the current pandemic caused by the virus responsible for COVID-19, further research into the OAF and the open-air regimen would appear to be essential. Unfortunately, both have a history of being overlooked, as Dr. Robert Saunby, a Professor of Medicine at Birmingham University, observed in 1914: 'Why have we been so slow to recognize that fresh air is the best tonic, the best antiseptic?' [50].

Implications for the future

Decades ago, hospitals and other building types were designed to prevent infections from spreading. High levels of natural ventilation were an absolute requirement. Today they are not. Fresh air is no longer considered to be germicidal or therapeutic for hospital patients or, for that matter, anyone else. Buildings are no longer designed for free access to it. For example, windows are smaller, ceilings are lower, cross-ventilation can be difficult if not impossible, and balconies and verandas are not as common as they once were. Yet, today there is a consensus that new respiratory pathogens increasingly threaten global public health. We also face the resurgence of old ones in more virulent forms. It is perhaps time to examine how we used to design and ventilate buildings for health. If this is ignored - just as the OAF continues to be - the costs to society could be large.

In order to move forward, we need:

• A program of testing both established and novel pathogens to determine the effects of the OAF on them.

• Experiments to determine whether, how best, and for how long the OAF can be preserved indoors.

• A review of building design with regard to improved infection control and patient recovery. This should focus on increased exposure and access to outside air and to the open-air factor.

## Conclusions

From the end of the 19th century to the middle of the 20th, there was a widely held belief that outdoor air had disinfecting and therapeutic properties. High volumes of fresh air were thought to be of fundamental importance to indoor health. The tall ceilings and big windows in schools, hospitals, offices, and domestic buildings of the period reflect this. The Nightingale ward and, subsequently, open-air sanatoria and military hospitals were arranged to exploit the germicidal effects of rural air and its putative therapeutic properties during the day and night. When the open-air regimen was superseded, the practice of designing hospitals and schools to this end was abandoned. During the 1960s, the germicidal properties of rural air were confirmed. As yet, we don't know how best the germicidal and health effects of outdoor air can be preserved indoors. Given the threat to global public health from COVID-19, antimicrobial-resistant bacteria, pandemic influenza, and novel pathogens, there is merit in investigating whether and how this can be done. If so, 'rediscovering' open-air wards and the open-air regimen might benefit patients and staff in hospitals. The OAF will likely also help in reducing the transmission of many infections in schools, homes, offices, and larger buildings.

A program of testing is needed to determine the effects of the OAF on the viability of established and emerging pathogens. Additional research should be carried out to confirm that OAF can be preserved indoors and under what conditions. Following this, a review of building design, with particular regard to infection control and patient recovery, should be undertaken as a matter of urgency. But we need to also recognize that there is already sufficient evidence to show that public health generally would improve if more emphasis was placed on increased exposure to outdoor air.
